# Metagenomic next-generation sequencing of samples from pediatric febrile illness in Tororo, Uganda

**DOI:** 10.1371/journal.pone.0218318

**Published:** 2019-06-20

**Authors:** Akshaya Ramesh, Sara Nakielny, Jennifer Hsu, Mary Kyohere, Oswald Byaruhanga, Charles de Bourcy, Rebecca Egger, Boris Dimitrov, Yun-Fang Juan, Jonathan Sheu, James Wang, Katrina Kalantar, Charles Langelier, Theodore Ruel, Arthur Mpimbaza, Michael R. Wilson, Philip J. Rosenthal, Joseph L. DeRisi

**Affiliations:** 1 Weill Institute for Neurosciences, University of California, San Francisco, California, United States of America; 2 Department of Neurology, University of California, San Francisco, California, United States of America; 3 Department of Biochemistry and Biophysics, University of California, San Francisco, California, United States of America; 4 Division of Infectious Diseases, Department of Medicine, University of California, San Francisco, California, United States of America; 5 Infectious Diseases Research Collaboration, Kampala, Uganda; 6 Chan Zuckerberg Biohub, San Francisco, California, United States of America; 7 Division of Pediatric Infectious Diseases and Global Health, Department of Pediatrics, University of California, San Francisco, California, United States of America; 8 Child Health and Development Centre, Makerere University, Kampala, Uganda; Defense Threat Reduction Agency, UNITED STATES

## Abstract

Febrile illness is a major burden in African children, and non-malarial causes of fever are uncertain. In this retrospective exploratory study, we used metagenomic next-generation sequencing (mNGS) to evaluate serum, nasopharyngeal, and stool specimens from 94 children (aged 2–54 months) with febrile illness admitted to Tororo District Hospital, Uganda. The most common microbes identified were *Plasmodium falciparum* (51.1% of samples) and parvovirus B19 (4.4%) from serum; human rhinoviruses A and C (40%), respiratory syncytial virus (10%), and human herpesvirus 5 (10%) from nasopharyngeal swabs; and rotavirus A (50% of those with diarrhea) from stool. We also report the near complete genome of a highly divergent orthobunyavirus, tentatively named Nyangole virus, identified from the serum of a child diagnosed with malaria and pneumonia, a Bwamba orthobunyavirus in the nasopharynx of a child with rash and sepsis, and the genomes of two novel human rhinovirus C species. In this retrospective exploratory study, mNGS identified multiple potential pathogens, including 3 new viral species, associated with fever in Ugandan children.

## Introduction

The evaluation of children with fever is challenging, particularly in Low and Middle income countries (LMIC). A febrile child in sub-Saharan Africa may have a mild self-resolving viral infection or may be suffering from bacterial sepsis or malaria—major causes of disability and death [1, 2]. Historically, febrile illness in much of Africa has been treated empirically as malaria due to the limited availability of diagnostics and the risk of untreated malaria progressing to life-threatening illness. This strategy changed in 2010 following revised guidelines from the World Health Organization (WHO), which recommended limiting malaria therapy to those with a confirmed diagnosis [3]. However, standard recommendations for management of febrile children who do not have malaria are lacking. Increased knowledge about the prevalence of non-malarial pathogens associated with fever is needed to inform management strategies for febrile children [2], especially in low resource settings.

Advances in genome sequencing hold promise for addressing global infectious disease challenges by enabling unbiased detection of microbial pathogens that can be used to design directed diagnostics, and improve surveillance in LMIC [4–5]. The unbiased approach to detection of sequence-based diagnostics have led to the successful detection of pathogens in some rare or complex cases where traditional methods have failed [6–10]. Sequence-based diagnostics are complementary to serological assays and may contribute to a better understanding of pathogen landscapes in LMIC. Towards this aim, we conducted an exploratory retrospective mNGS analysis on samples available from a cohort of children hospitalized in rural Uganda with febrile illnesses to characterize potential pathogens associated with fever. The results, which include the detection of 3 novel viral species, suggest that mNGS will likely be a valuable tool in the arsenal of assays to understand the microbial landscape in human infections.

## Results

### Clinical characteristics of subjects with febrile illness

From October to December 2013, 94 children admitted to Tororo District Hospital were enrolled (Table 1). Their mean age was 16.4 (IQR: 8.0–21.0) months, and 66 (70.2%) were female. Chief symptoms reported in addition to fever were cough (88.3%), vomiting (56.4%), diarrhea (47.9%), and convulsions (27.7%). Top admitting diagnoses were respiratory tract infection (57.4%), gastroenteritis/diarrhea (29.8%), and septicemia (11.7%) (Table A in S1 Data). Of the 90 blood samples that were collected, thick blood smears identified *P*. *falciparum* in 12 samples that underwent mNGS analysis (Table B in S1 Data).

**Table 1:** Overview of the patients enrolled in the study. The other category includes: unknown (10), urinary infection (1), meningitis (2), hepatitis (1), and fever (1). Information on gender was missing for three and on age for two children.

Clinical category (number of patients in category)	Age (mean, months)	Gender	
		Male	Female
Respiratory illness (54)	14.0	25	28
Diarrhea/gastroenteritis (28)	12.1	9	19
Malaria (11)	15.5	3	7
Sepsis (11)	19.9	5	6
Malnutrition (5)	18.4	2	3
Other (15)	21.1	11	3

### Metagenomic sequencing findings

mNGS was performed on RNA extracted from 90 serum, 90 NP swab, and 10 stool samples following library preparation. A mean of 11.5 million (IQR 6.4–15.2 million) paired-end reads were obtained per sample; sequencing statistics are in S2 Data. For one batch of serum samples, only a single read, rather than paired-end reads, was produced. Bioinformatic analysis was carried out using the IDseq pipeline, a cloud-based, open-source platform designed for detection of microbes from metagenomic data (https://github.com/chanzuckerberg/idseq-dag) that incorporates several features of previously developed pathogen detection pipelines [11–17].

In this section, we discuss the mNGS results identified in a given sample type. Detailed findings on microbes identified in every patient, along with the total reads per million (rpM) are reported in Table B and Table C in S1 Data, respectively.

### mNGS of serum

**Fig 1:**
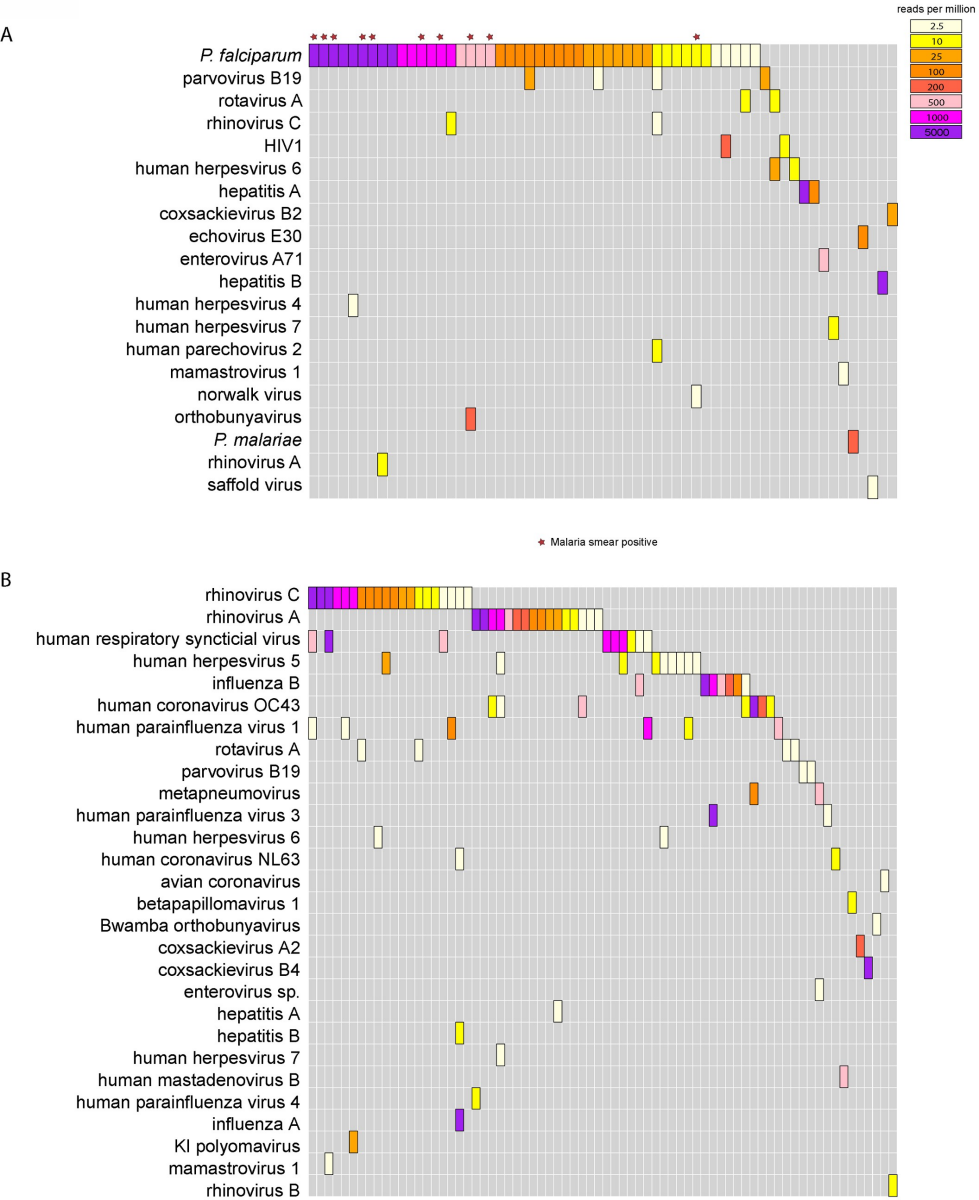
**(A) Microbial landscape found in serum samples in Ugandan children.** Each column represents a febrile child. Results for GB virus C and torque teno virus, which are of uncertain clinical significance, are not included. **(B) Microbial landscape found in nasopharyngeal (NP) swab samples in Ugandan children.** Note that bacterial species were not considered in Fig 1B. Each column represents a febrile child, and the color bars represent the total reads per million (rpM) of a particular microbe present in the sample. Results for GB virus C and torque teno virus, which are of uncertain clinical significance, are not included.

At least one microbial species was detected in 60 (66.7%) of the serum samples; more than one microbe was detected in 11 (12.2%) samples (Fig 1A). No microbial species were identified in the serum of 30 (33.3%) individuals. The most commonly identified microbes were *Plasmodium falciparum* (46, 51.1%) and parvovirus B19 (4, 4.4%). *P*. *falciparum* was detected in 10/12 samples from patients reported as smear-positive. mNGS detected *Plasmodium spp*. in 37 additional samples that were smear negative (36 *P*. *falciparum*, 1 *P*. *malariae*). Viruses detected in serum included human immunodeficiency 1 virus (HIV-1), hepatitis A virus, rotavirus A, human herpesvirus (HHV) type 6, HHV type 4, HHV type 7, human rhinovirus (HRV)-C, HRV-A, enteroviruses (enterovirus A71, Coxsackievirus B2 and echovirus E30), human parechovirus 2, hepatitis B virus, a novel orthobunyavirus (described in greater detail below), human cardiovirus (Saffold virus), mamastrovirus 1 and Norwalk virus (Fig 1A).

Multiple viruses were detected from serum in patients with *Plasmodium* infections (10 of 46 (21.7%) samples; S1A Table). Three of the four identified parvovirus B19 cases were associated with *P*. *falciparum*. Additionally, GB virus C and torque teno virus (TTV), which are of unknown clinical significance [18], [19], were identified in the serum of 25 (27.8%) and 37 (41.1%) children, respectively. There have been reports on associations between immunosuppression and TTV abundance [20, 21]. Interestingly, a prior study has reported a higher abundance of TTV in children with fevers [22].

### mNGS of NP swabs

90 NP swabs were collected and processed; 52 (57.7%) of these were from patients with admission diagnoses of pneumonia, respiratory tract infection, or bronchiolitis (Table 1). Chest imaging was not available to further assess these diagnoses. 72 NP samples (80%) contained at least 1 viral species (Fig 1B), with no microbes meeting our required cut-offs in 18 samples (20%). HRV-A and HRV-C were the most prevalent, followed by respiratory syncytial virus (RSV), cytomegalovirus (HHV-5), influenza B, and coronavirus OC43. Other respiratory viruses identified included influenza A (H1N1), HRV-B, Human mastadenovirus B (type 7), three human parainfluenza virus types (type 1, 3 and 4), metapneumovirus, coronavirus NL63, avian coronavirus, coxsackievirus A2, coxsackievirus B2, polyomaviruses (KI), HHV-6 and HHV-7. Other viruses identified that are not typically considered respiratory pathogens included hepatitis A virus, hepatitis B virus, parvovirus B19, mamastrovirus 1, Bwamba orthobunyavirus, betapapillomavirus 1, and rotavirus. Additionally, TTV was found in 49 (54.4%) NP swab samples, including one sample with both gemykrogvirus and TTV. For 26 (28.8%) patients, mNGS identified respiratory viral co-infections, most commonly with HRV-C (n = 11) and HRV-A (n = 5) (S1B Table). The same microbial species was identified in the NP swab and serum samples in six patients (on independent sequencing runs), one each with HRV-A, HRV-C, hepatitis A virus, hepatitis B virus, rotavirus A, and parvovirus B19.

Bacteria identified in NP samples included four dominant genera, which together comprised 79% of all microbial reads—*Moraxella* (39.4%), *Haemophilus* (16.7%), *Streptococcus* (16.2%), and *Corynebacterium* (6.6%). Given that diversity loss in the microbial flora in lower respiratory tract samples correlates with pneumonia [23, 24], we compared the Simpson Diversity Index (SDI) in patients with and without clinical diagnoses of respiratory tract infection. We found no significant difference in the SDI of upper airway samples between patients with (mean SDI = 0.51, IQR 0.37–0.65) or without (mean SDI = 0.51, IQR = 0.42–0.65; p = 0.86) diagnoses of respiratory infection (S1 Fig). [25–27].

### mNGS of stool

Among the 10 stool samples collected and sequenced, potential non-bacterial pathogens were detected in 9/10 samples. The three most common microbes identified were rotavirus A (50%), *Cryptosporidium* (40%), and human parechovirus (40%). Our sequencing did not provide enough information to type the identified human parechovirus. Seven children had additional microbes identified: two rotavirus A and human parechovirus, and one each rotavirus A and *Cryptosporidium*, rotavirus A and enterovirus, *Cryptosporidium* and human parechovirus, *Cryptosporidium* and Norwalk virus, and *Giardia* and human parechovirus. Five samples also had *Blastocystis hominis*, a protozoan of uncertain pathogenicity.

### Genomic characterization of viruses

Representative genomes for all viruses identified in this study were assembled and deposited in GenBank (Accession numbers: MH685676-MH685701, MH685703- MH685719, MH684286-MH684293, MH684298-MH684334). In this section, we describe in detail a novel orthobunyavirus identified in the serum of one individual, two novel HRV-C species identified in two individuals and diversity of influenza B viruses assembled in the nasopharynx from five individuals. We studied genomic diversity of influenza B in detail, given its potential implication to inform vaccine design. We did not include influenza A in this analysis, as we were able to assemble the complete genome sequence only from one individual.

### Orthobunyaviruses

**Fig 2:**
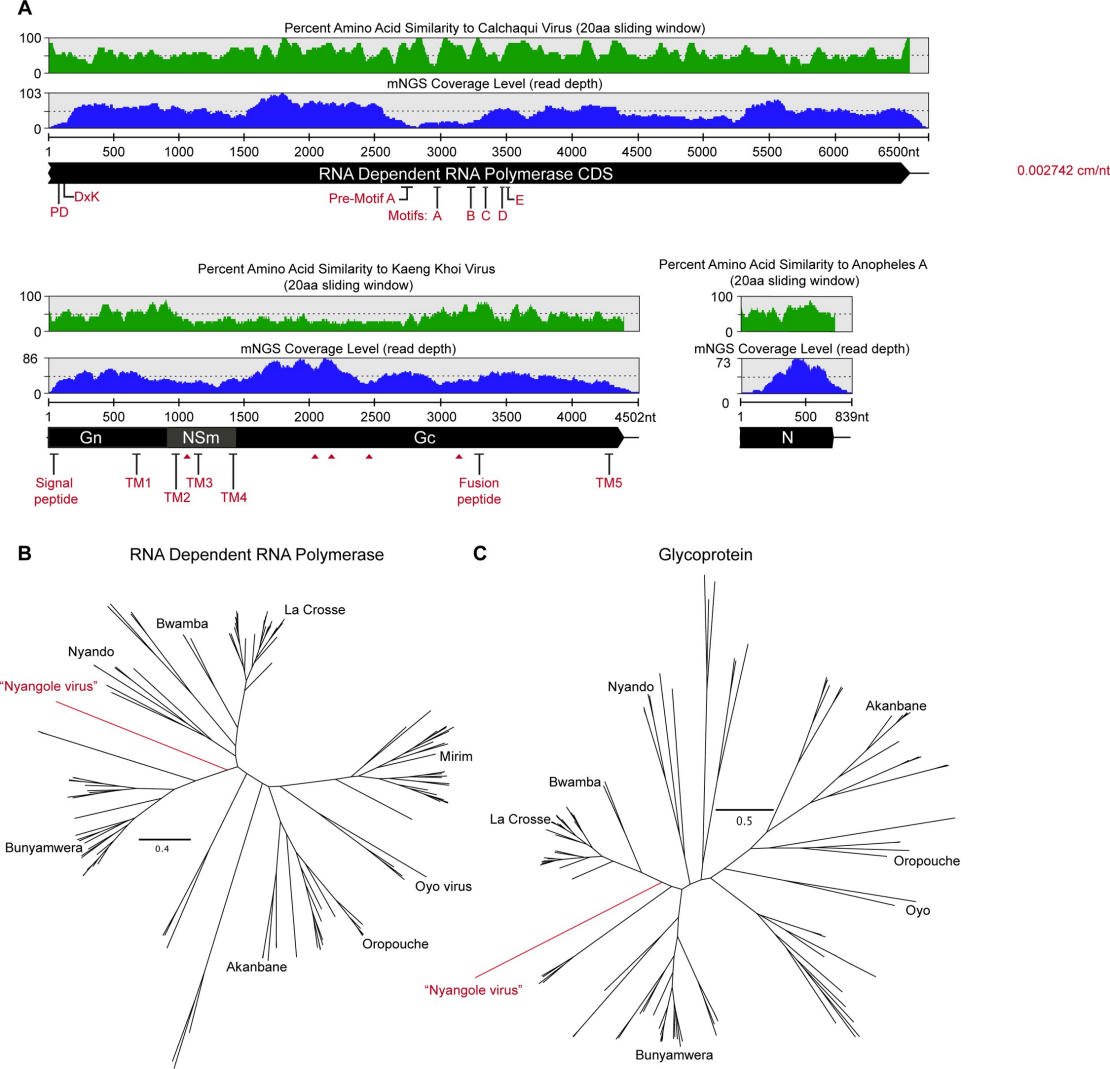
Characterization of the novel orthobunyavirus identified in a febrile child. (A) Schematic representation of the large (L) or RNA dependent RNA polymerase, medium (M) or polyprotein of Gn, NSm and Gc proteins and small (S) segment encoding the nucleocapsid (N) protein of Nyangole virus and percentage identity with the most closely related virus. Phylogenetic tree of all complete orthobunyavirus genome sequences along with Nyangole virus are represented in (B) for the RNA dependent RNA polymerase and (C) for the glycoprotein.

Serum from one patient admitted with a clinical diagnosis of malaria and pneumonia contained a novel orthobunyavirus in addition to *P*. *falciparum*. Assembly of a near-complete genome and comparison with existing orthobunyavirus genomes indicated that this sequence includes 97.5%, 100% and 91% of the L, M, and S coding regions, respectively (Fig 2). Average read coverage across the segments was 86-fold. Phylogenetic comparison showed that the novel virus was significantly divergent from known orthobunyaviruses, sharing 44.9–55.1% amino acid identity with the closest known relatives, Calchaqui virus, Kaeng Khoi virus, and Anopheles A virus (Figs 2 and S2, S3 and S4). The virus was isolated from a patient from Nyangole village, Tororo District—hence, we propose the name “Nyangole virus”, consistent with nomenclature guidelines for the family *Bunyaviridae*.

In addition, a second orthobunyavirus, Bwamba virus, was identified in the NP swab sample from a patient admitted with rash, sepsis, and diarrhea. Insufficient sample and sequencing reads precluded genome assembly of this virus.

### Human rhinoviruses

**Fig 3:**
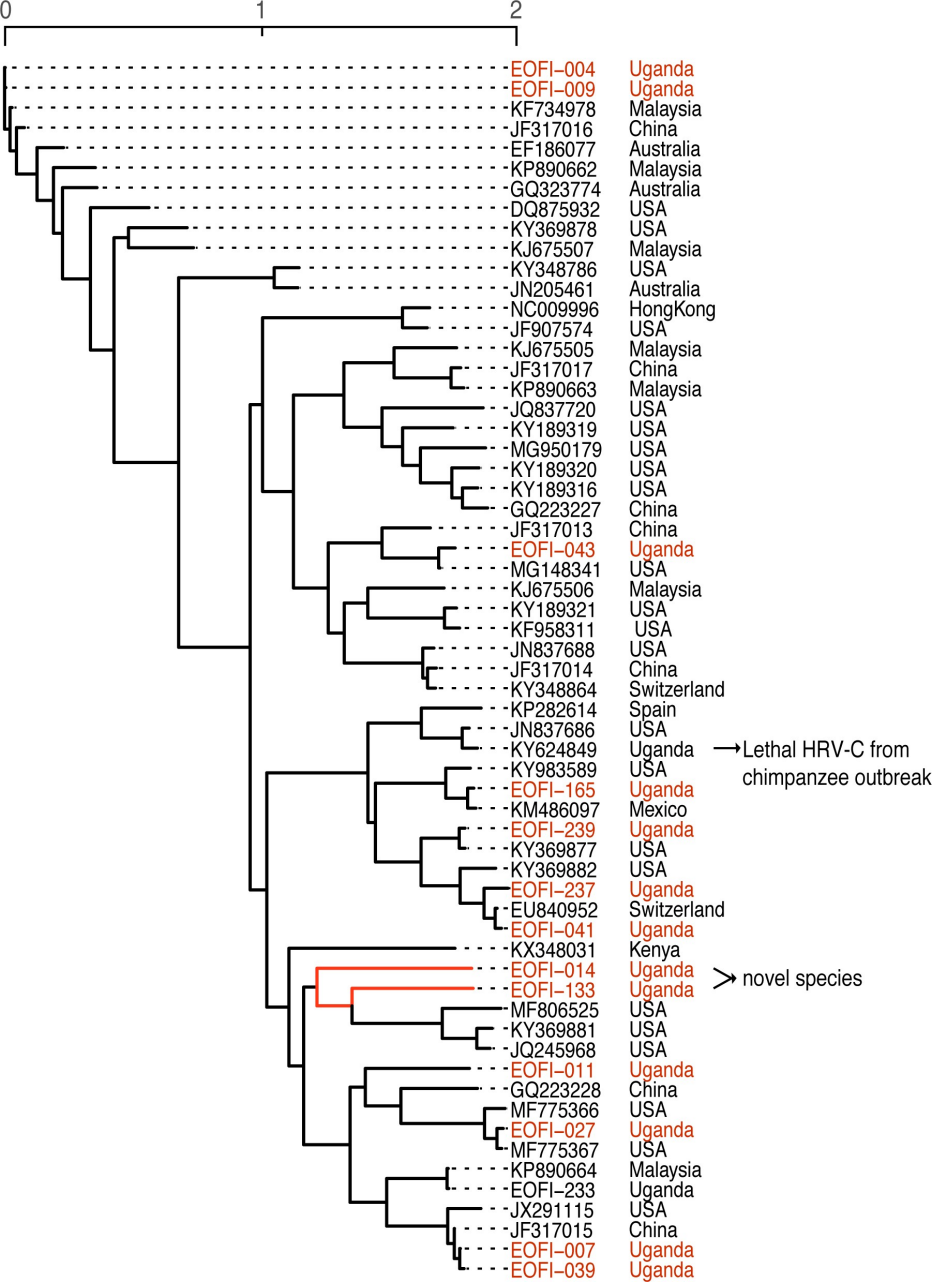
Phylogenetic tree of all complete HRV-C genomes from NCBI (with information on country of isolation) and HRV-C genomes assembled in this study.

Within the species rhinovirus, we assembled *de novo* a total of 13 HRV-C (mean coverage: 39-fold) and 13 HRV-A (mean coverage: 268-fold) genomes (> 500 bp). Of these, 10 HRV-A and nine HRV-C genomes had complete coverage of the VP1 region, which is used to define enterovirus types [28]. Unique HRV types are defined by <73% similarity in the VP1 gene. As such, we found three HRV-A and eight HRV-C types in this cohort. One individual harbored two distinct HRV-A types (genome pairwise identity = 75.3%, VP1 pairwise identity = 67.1%). Additionally, we assembled two novel HRV-C species from two patients admitted with gastroenteritis (patient ID: EOFI-014) and with pneumonia, malaria and diarrhea (patient ID: EOFI-133), that shared 70.1% and 70.7% nucleotide sequence identity at VP1 compared to the closest known HRV-C (Accession JQ245968 and KF688606, respectively) (Fig 3). The Picornavirus Working Group has established that novel HRV-Cs should exhibit at least 13% nucleotide sequence divergence in the VP1 gene [29], qualifying these two as novel.

### Influenza B virus

We assembled influenza B genome segments (>500bp, mean-coverage: 4-fold) from six of seven samples containing influenza B virus (one sample had insufficient sequencing reads). The viruses assembled were >99% similar to each other and >99% identical to the B/Massachusetts/02/2012-like virus included in the vaccine recommended by WHO for the 2013–2014 northern hemisphere and 2014 southern hemisphere influenza seasons (Accession numbers: KC891816.1, KC891879.1 and KC892119.1). For the Ugandan viruses, three of the four major epitopes (150 loop, 160 loop and 190 helix) and their surrounding regions were 100% identical; a Ser->Thr substitution was observed at amino acid 136 (in the 120 loop) compared to the B/Massachusetts/02/2012-like virus.

## Discussion

A better understanding of the microbial agents causing fever in African children is needed to inform the development of better diagnostic algorithms, therapeutic guidelines and public health strategies. We performed an exploratory retrospective study with unbiased mNGS on various tissue types to determine whether this technology has potential to contribute to our understanding of the etiologies of fever in African children. In this limited sample set, mNGS identified a wide range of potential pathogens, including three novel viral species.

Other studies evaluating causes of febrile illness in African children have focused on a limited number of pathogens [30–33]. In a study of febrile children in Tanzania utilizing serologic, culture, and molecular assays, viruses accounted for 51% of lower respiratory infections, 78% of systemic infections, and 100% of upper respiratory infections [34]. Additionally, in the above study, 9% of the children had malaria and 4.2% had bacteremia. In febrile children in Kenya, reported pathogens were spotted fever group *Rickettsiae* (22.4%), influenza (22.4%), adenovirus (10.5%), parainfluenza virus 1–3 (10.1%), Q fever (8.9%), RSV (5.3%), malaria (5.2%), scrub typhus (3.6%), human metapneumovirus (3.2%), group A *Streptococcus* (2.3%) and typhus group Rickettsiae (1.0%) [35, 36]. Another study reported bacteremia in 19.1% of children admitted to a referral hospital in Uganda [37]. Additionally, in patients (across all age groups) with severe febrile illness, bacteremia was detected in 10.1% in North Africa, 10.4% in East Africa, and 12.4% in West Africa [38]. In this small study, *Plasmodium falciparum* was identified in the serum of 51.1% of the children, human rhinoviruses A and C dominated in the nasopharyngeal swab of 40% of the children and rotavirus A was identified in the stool samples of 50% of the children studied. For 20% of NP swabs and 33.3% of serum samples, no microbial species met our thresholds for detection. These proportions are consistent with previous reports [32, 34, 35, 39].

Unbiased sequencing approaches are designed to identify all potential pathogens but have also been limited by high cost and infrastructure needs. Given the exploratory nature of this mNGS study, we cannot ascertain population level incidence or prevalence of particular infections. As expected, in the serum samples, *P*. *falciparum* was most commonly identified [40]. Some discrepancies were seen compared to blood smear readings, with false positive smears probably due to errors in slide reading, a common problem in under resourced clinics [41], and false negative smears due to the expected greater sensitivity of mNGS for identification of *P*. *falciparum*. In children with only sub-microscopic parasitemia, it is uncertain whether fevers can be ascribed to malaria, and in fact many children had both *P*. *falciparum* and additional microbes identified. Interestingly, three of the four cases of parvovirus B19 were found in association with *P*. *falciparum*; this co-infection has been associated with severe anemia with life-threatening consequences [42–44].

For NP and stool samples, given that the nasopharynx and intestines are normally colonized with commensal bacteria [45–48], and the lack of samples from healthy Ugandan controls, we focused on non-bacterial species. HRV was the most commonly identified virus in NP swab samples, consistent with findings previously reported in sub-Saharan Africa and developed countries [49–53]. HRV-C was most frequently encountered (54.1%), followed by HRV-A (43.2%) and HRV-B (2.7%), similar to the distribution of HRVs previously reported in Kenya [49]. We identified two novel HRV-C species which were approximately 70% identical to the most closely related previously described HRV-C species [29]. Overall, we detected at least three HRV-A and eight HRV-C types co-circulating in Tororo District. Of note, during the same collection period, a lethal HRV-C outbreak was reported in chimpanzees in Kibale National Park, in western Uganda [54]; that HRV-C was modestly related to an isolate observed in our study (74% nucleotide identity; 81% amino acid identity) (Fig 3) [54]. Our results confirm that a wide spectrum of HRVs infects Ugandan children. In addition to HRV, we detected a number of other known respiratory viruses, including RSV, human parainfluenza viruses, human coronaviruses, and adenovirus.

Diarrheal disease is one of the leading causes of death in children in Africa [55]. Approximately 48% of febrile children in our study presented with diarrhea, but due to logistical constraints stool specimens were available for only 10 cases. Rotavirus A, the leading cause of pediatric diarrhea worldwide [56], was the most commonly identified microbe in this cohort. Rotavirus vaccination, known to be highly effective, is yet to be implemented in Uganda, but the need is clear [56]. In addition to rotavirus A, we detected *Cryptosporidium*, norovirus, *Giardia*, *B*. *hominis* and several enteroviruses in stool specimens. Enteroviruses, HRV-C, and mamastrovirus were also identified in the serum of three children with clinical diagnoses of gastroenteritis or diarrhea.

Unbiased inspection of microbial sequences from sera revealed a novel member of the orthobunyavirus genus, tentatively named Nyangole virus, which was identified along with *P*. *falciparum* in a child with clinical diagnoses of malaria and pneumonia. The virus was surprisingly divergent from known viruses, with an average amino acid similarity of 51.6% to its nearest known relatives, including Calchaqui, Anopheles A and Kaeng Khoi viruses. Mosquitoes have been proposed as a vector for Calchaqui and Anopheles A viruses; Kaeng Khoi virus has been isolated from bedbugs [57–59]. Antibodies to these viruses have been detected in human sera, but their role as human pathogens is uncertain [46–49]. However, other orthobunyaviruses are responsible for severe human illnesses (e.g., Oropouche, Bunyamwera virus, California encephalitis virus, La Crosse virus, Jamestown Canyon virus, and Cache Valley virus) [60]. While the coverage depth of the assembled Nyangole virus genome in our patient suggests significant viremia, it is unknown whether the identified virus was responsible for the patient’s febrile illness.

NP swab analysis identified another orthobunyavirus, Bwamba virus, in a child admitted with rash, sepsis and diarrhea. This virus has previously been described to cause fever in Uganda [61]. Our identification of two orthobunyaviruses, including one novel virus, in a small sample of febrile Ugandan children suggests that the landscape of previously unidentified viruses that potentially infect African children and potentially cause febrile illness, is significantly under explored.

In addition to pathogen identification, the capacity of mNGS to provide viral strain resolution suggests its utility for monitoring vaccine efficacy by assessing prevalence of vaccine-targeted versus non-targeted strains. In the case of influenza B virus, the WHO recommended vaccine for 2013/2014 was highly conserved to the virus present in Uganda during that season.

Our exploratory pilot study had important limitations. First, our samples were not collected randomly, but rather were a retrospective convenience sample due to logistical constraints; as such, the results are not necessarily representative of pathogens infecting Ugandan children. In particular, the lack of identification of bacteremia in study subjects may have been due to a relative paucity of severe illness, compared to that in other studies. Second, the samples were collected only over a period of three months (October—December). Hence, we are unable to comment on seasonal trends in identified pathogens. Third, clinical evaluation of children followed the standards of a rural African hospital, so diagnostic evaluation was limited to physical examination and malaria blood smears. This study was not designed to compare mNGS to other clinical or laboratory assays. It is clear that more will be learned by linking rigorous clinical evaluation with mNGS results, and thereby more comprehensively assessing associations between clinical syndromes and specific pathogens. Fourth, healthy controls from the same population were not recruited in this study, hence we were unable to include them in the background model to filter out commensal microbial species specific to the Ugandan microbiome. Fifth, we were unable to use orthogonal techniques such as PCR to confirm the microbial species identified by mNGS due to lack of sample availability.

Given these limitations, we hesitate to integrate all the clinical specimens on a per sample basis, and rather present a portrait of all the microbes identified in febrile children. For readers interested in a breakdown of all microbial species from all samples collected per child, Table B in S1 Data contains this information. Future metagenomic studies should include rigorous clinical and microbiological phenotyping, along with samples collected from healthy individuals. This would facilitate design of an appropriate background model to identify potential pathogens, with confirmation using orthogonal techniques. Despite these limitations, our study provides an important snapshot of causes of fever in African children that could not be identified by available diagnostics, and suggests mNGS will be an important tool for future investigations. Given the yield of novel species in this small study alone, it is likely that an expanded use of this approach will continue to yield an increasingly rich portrait of microbial diversity associated with disease in this region.

## Methods

This study was approved by the Makerere University Research and Ethics Committee, the Uganda National Council of Science and Technology, and the University of California, San Francisco Committee on Human Research. Written informed consent was obtained from the parent or guardian on the child's behalf for all child participants enrolled in this study.

### Enrollment of study subjects

We studied children admitted to Tororo District Hospital, Tororo, Uganda, with febrile illnesses. Potential subjects were identified by clinic staff, who notified study personnel, who subsequently evaluated the children for study eligibility. Inclusion criteria were: 1) age 2–60 months; 2) admission to Tororo District Hospital for acute illness; 3) documentation of axillary temperature >38.0°C on admission or within 24 hours of admission; and 4) provision of informed consent from the parent or guardian for study procedures. The only exclusion criterion was unwillingness or inability of parents/guardians to provide consent.

### Sample collection

Serum and nasopharyngeal (NP) swab samples were collected from 90 children each; for four children, only one of the two sample types was successfully collected. Although 45 (47.9%) of the children had a presenting symptom of diarrhea, stool samples were available for only 10 due to logistical constraints. All samples that were collected were processed and included in the analysis.

### Study specimens

NP swabs and serum were collected from each enrolled subject within 24 hours of hospital admission. Approximately 5 ml of serum was collected by phlebotomy, the sample was centrifuged at room temperature, and serum was then stored at -80°C. NP swab samples collected with FLOQSwabs swabs (COPAN) were placed into cryovials with Trizol (Invitrogen), and stored at -80°C within ~5 min of collection. For subjects with acute diarrhea (≥ three loose or watery stools in 24 hours), stool was collected into clean plastic containers and stored at -80°C within ~5 min of collection. Samples were stored at -80°C until shipment on dry ice to UCSF for sequencing.

### Clinical data

Clinical information was obtained from interviews with parents or guardians, with specific data entered onto a standardized case record form that included admission diagnosis and physical examination as well as malaria blood smear results. For malaria diagnosis, thick Blood smears were Giemsa stained and evaluated by Tororo District Hospital laboratory personnel following routine standard-of-care practices. No efforts were made to improve on routine practice, so malaria smear readings represent routine standard-of-care rather than optimal quality controlled reads.

### Metagenomic next-generation sequencing (mNGS)

After shipment to University of California, San Francisco, RNA was extracted from clinical samples as well as positive (HeLa cells) and negative (water) controls, and unbiased cDNA libraries were generated using previously described methods (see sections “Sequencing library preparation” and “Metagenomic Library Preparation”, respectively) [62, 5]. Barcoded samples were pooled, size selected (Blue Pippin), and run on an Illumina HiSeq2500 to obtain 135 base pair (bp) paired-end reads.

### Bioinformatic analysis and pathogen identification

Microbial pathogens were identified from raw sequencing reads using the IDseq (v1.6) Portal (https://idseq.net), a cloud-based, open-source bioinformatics platform designed for detection of microbes from metagenomic data. IDseq is a scalable and cloud based implementation of a previously published pipeline (see Fig 1 in [11]). In brief, initial host read filtering is performed using Spliced Transcripts Alignment to a Reference (STAR) algorithm [17], followed by removal of duplicate/low quality and low complexity sequences [14], [16]. Next, reads are aligned once again to the host genome of interest using bowtie2 [12] to remove any remaining host reads. The non-human reads are then aligned to the NCBI nucleotide and protein database, using GSNAPL and RAPSearch, respectively [13], [15]. Additionally, reads that were identified as HHV-5 were assessed individually using BLAST, to verify specificity to this virus. All IDseq scripts and user instructions are available at https://github.com/chanzuckerberg/idseq-dag and the graphical user interface web application for sample upload is available at https://github.com/chanzuckerberg/idseq-web. To distinguish potential pathogens from ubiquitous environmental agents including laboratory reagent contaminants and skin commensal flora, a Z-score was calculated for both nucleic acid and protein alignments, for each genus relative to a background of non-templated (“water only”) controls in addition to a previously published set of uninfected clinical mNGS samples [11]. CSF samples acquired through lumbar puncture from uninfected controls are included in this background model as there is extensive representation of the skin microbiome given the need to puncture the skin. Only human papillomavirus was identified in the positive (HeLa cell) controls.

For this study we report species greater than 0 reads per million (rpM) and a Z-score > 0 (for both nucleic acid and protein alignments) detected in the serum, stool, and NP samples. We chose to report all species satisfying these criteria, rather than restricting to particular species, to offer an unbiased representation of microbes present in the sample. IDseq uses the CD-HIT-DUP tool to compress duplicate reads, hence final assignment of the rpM in a given sample represents coverage across the genome, rather than a single portion. Consistent with previous studies, low levels of “index bleed through” or “barcode hopping” (assignment of sequencing reads to the wrong barcode/index) was observed within the non-templated water control samples [63]. To account for barcode mis-assignment, when a microbe was found in more than one sample, it was reported only when present at levels at least four times the level of mis-assigned reads observed in the control samples. Given the extremely high levels of rotavirus found in stool samples, these samples were run in duplicate, and only microbes identified in both replicates and present at levels at least four times the number of reads mis-assigned in the control samples were reported. If the reads identified for a given microbe were not species-specific, we reported the corresponding genus. For NP and stool samples, because the nasopharynx and intestines are normally colonized with commensal bacteria [45–48], and because of a lack of healthy Ugandan NP and stool samples to serve as controls, only non-bacterial species were reported though we did analyze NP microbiome diversity (see below).

### Genome assembly, annotation and phylogenetic analysis

To more comprehensively characterize the genomes of identified microbes, the paired-read iterative contig extension (PRICE) assembler [16] and the St. Petersburg genome assembler (SPAdes) [64] were used to *de novo* assemble short read sequences into larger contiguous sequences (contigs). Assembled contigs were queried against the National Center for Biotechnology Information (NCBI) nucleotide (nt) database using the basic local alignment search tool (BLAST) to identify the closest related microbes. GenBank annotation files from genome sequence records corresponding to the highest scoring alignments were used to identify potential features within the *de novo* assembled genomes. Geneious v10.3.2 was used to annotate newly assembled genomes. Reference genomes for multiple sequence alignments and phylogenetic analyses were downloaded from NCBI. Multiple sequence (nucleotide) alignments were generated using the default settings in MUSCLEv3.8.1551 [65], and ModelTest-NGv0.1.5 was used to identify the best-fitting evolutionary model. Using the best-fitting model of for evolution, we reconstructed a maximum-likelihood phylogeny using RAxML-ng v0.6.0 using default settings [66]. Annotation of protein domains in the novel orthobunyavirus was performed using the InterPro webserver [67] as well as direct alignment against previously known orthobunyaviruses. The TOPCONS webserver [68] was used for the identification of transmembrane regions and signal peptides, and the NetNglyc 1.0 Server (http://www.cbs.dtu.dk/services/NetNGlyc/) for the identification of glycosylation sites.

### Evaluation of NP microbiome diversity

We applied SDI to evaluate alpha diversity of microbes identified in NP samples. For this analysis, patients were stratified into two categories based on clinical assignment: respiratory infections (admitting diagnosis of pneumonia, respiratory tract infection, or bronchiolitis; n = 52) and all other syndromes (n = 39); cases with unknown admitting diagnosis were excluded. SDI was calculated in R using the Veganv2.4.4 package on genus-level reads per million values for all microbes, including bacteria. A Wilcox Rank Sum test was used to evaluate differences in SDI between patients in the two categories.

## Supporting information

S1 Fig Simpsons diversity index (SDI) for samples with pneumonia versus other etiologies. Each triangle represents one sample.
(TIF)

S2 Fig Complete phylogenetic tree of Large (L) or RNA dependent RNA polymerase. (TIF)

S3 Fig Complete phylogenetic tree of Medium (M) segment polyprotein encoding Gn, NSm and Gc proteins. (TIF)

S4 Fig Complete phylogenetic tree of Small (S) segment encoding Nucleocapsid segments. (TIF)

S1 Table (A) Co-infection table for *P*. *falciparum*, (B) Co-infection table for HRV.
(DOCX)

S1 Data (A) Admission categories of patients enrolled in the study. (B) mNGS findings in patients enrolled in the study. (C) Total rpM identified of microbial species in the serum, NP swab and stool.
(XLSX)

S2 Data Total number of sequencing reads and unique non-human reads in all samples analyzed. (XLSX)
