# Supplementary figures and images for: Metagenomic next-generation sequencing of samples from pediatric febrile illness in Tororo, Uganda
Source: PLoS One. 2019 Jun 20;14(6):e0218318. doi: 10.1371/journal.pone.0218318 (PMC6586300; doi:10.1371/journal.pone.0218318)

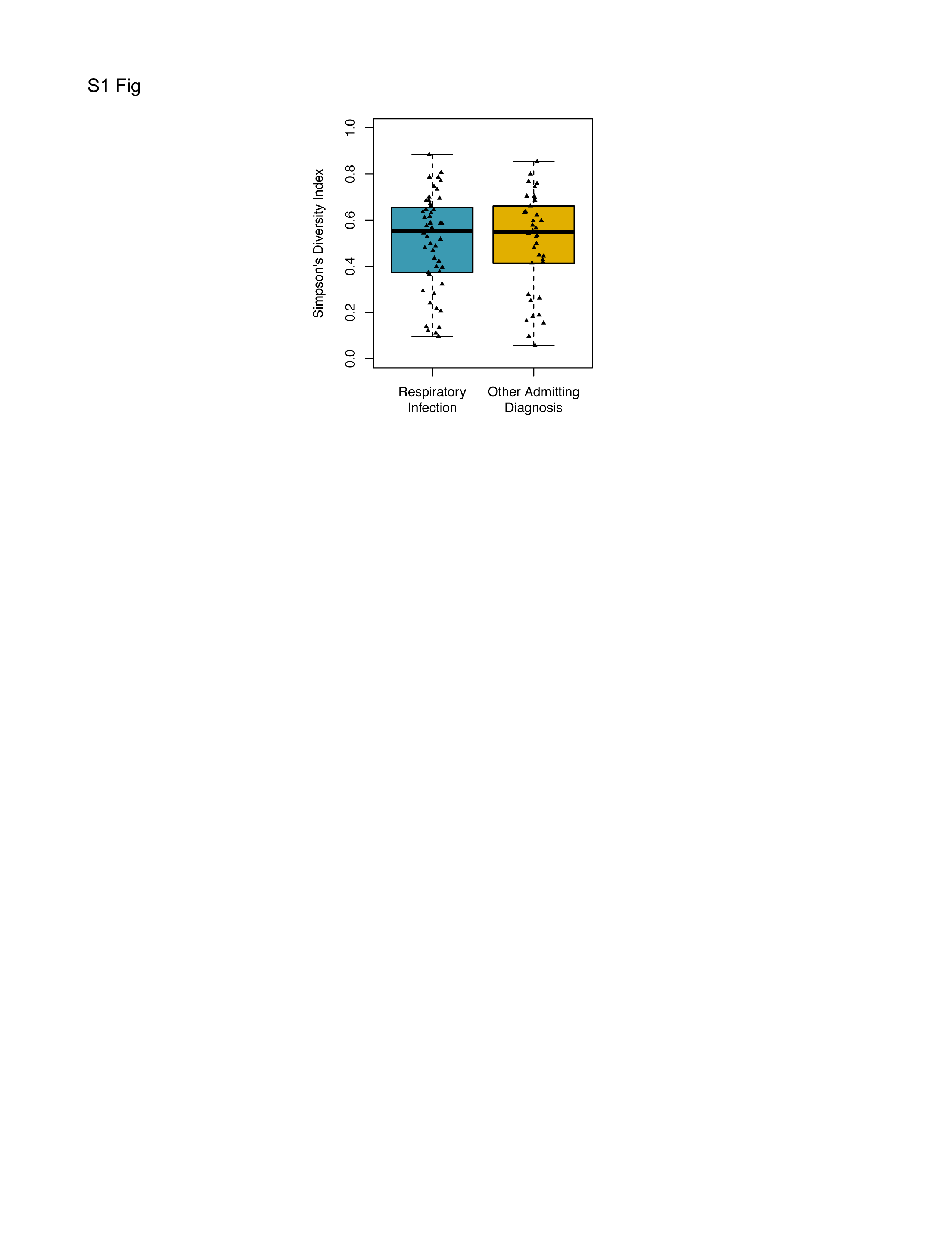

Supplement: S1 Fig — Each triangle represents one sample. (TIF) [file pone.0218318.s001.tif]

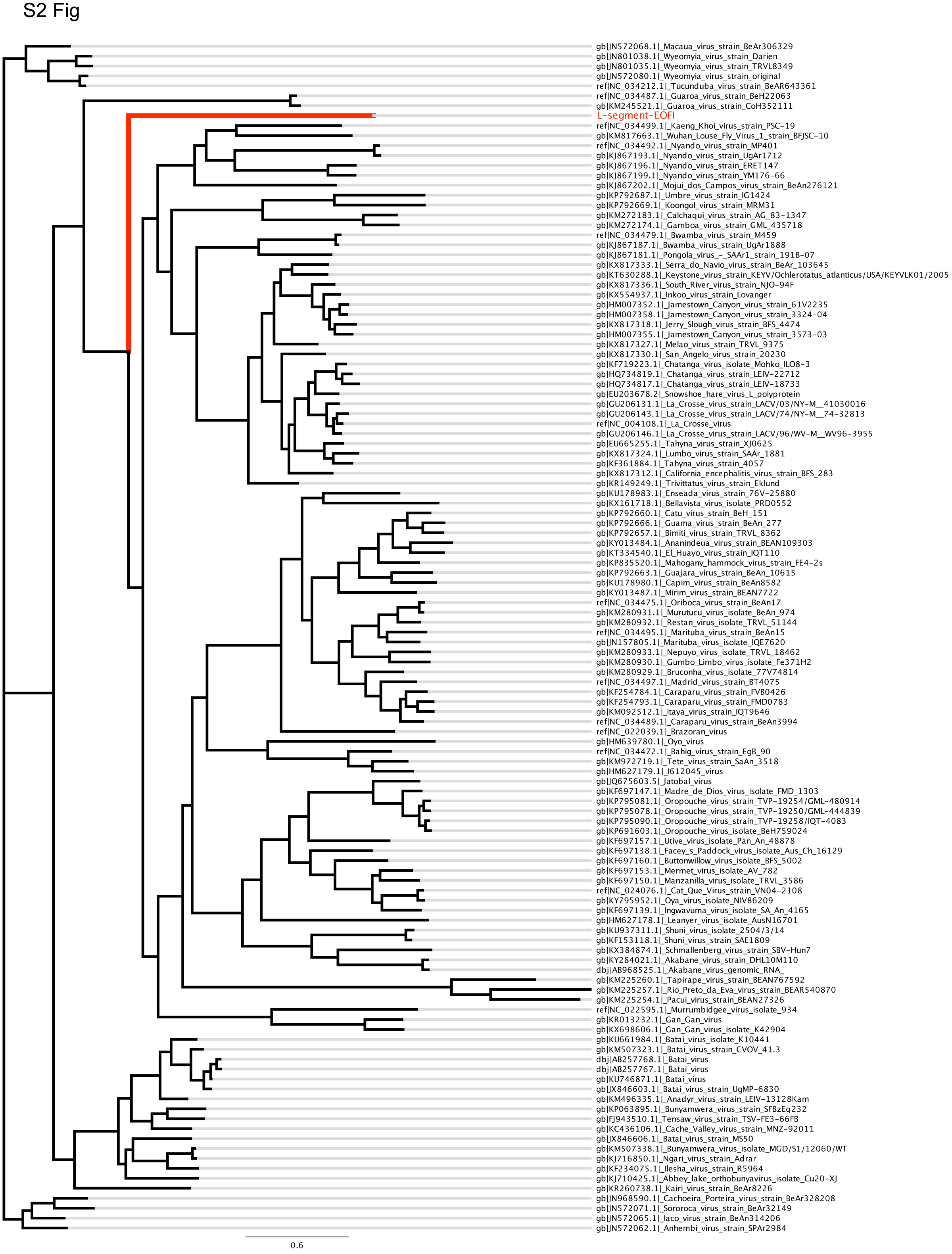

Supplement: S2 Fig — (TIF) [file pone.0218318.s002.tif]

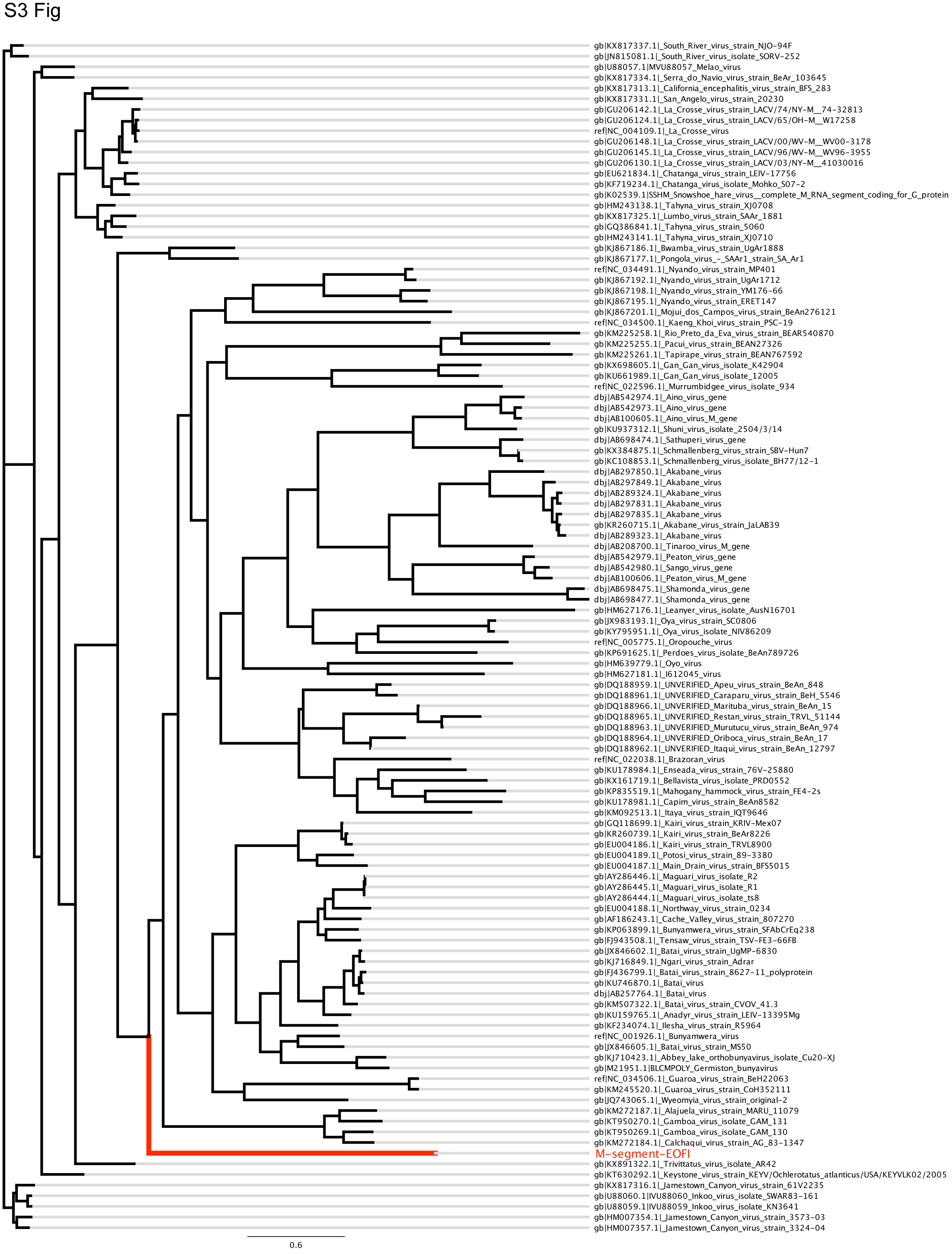

Supplement: S3 Fig — (TIF) [file pone.0218318.s003.tif]

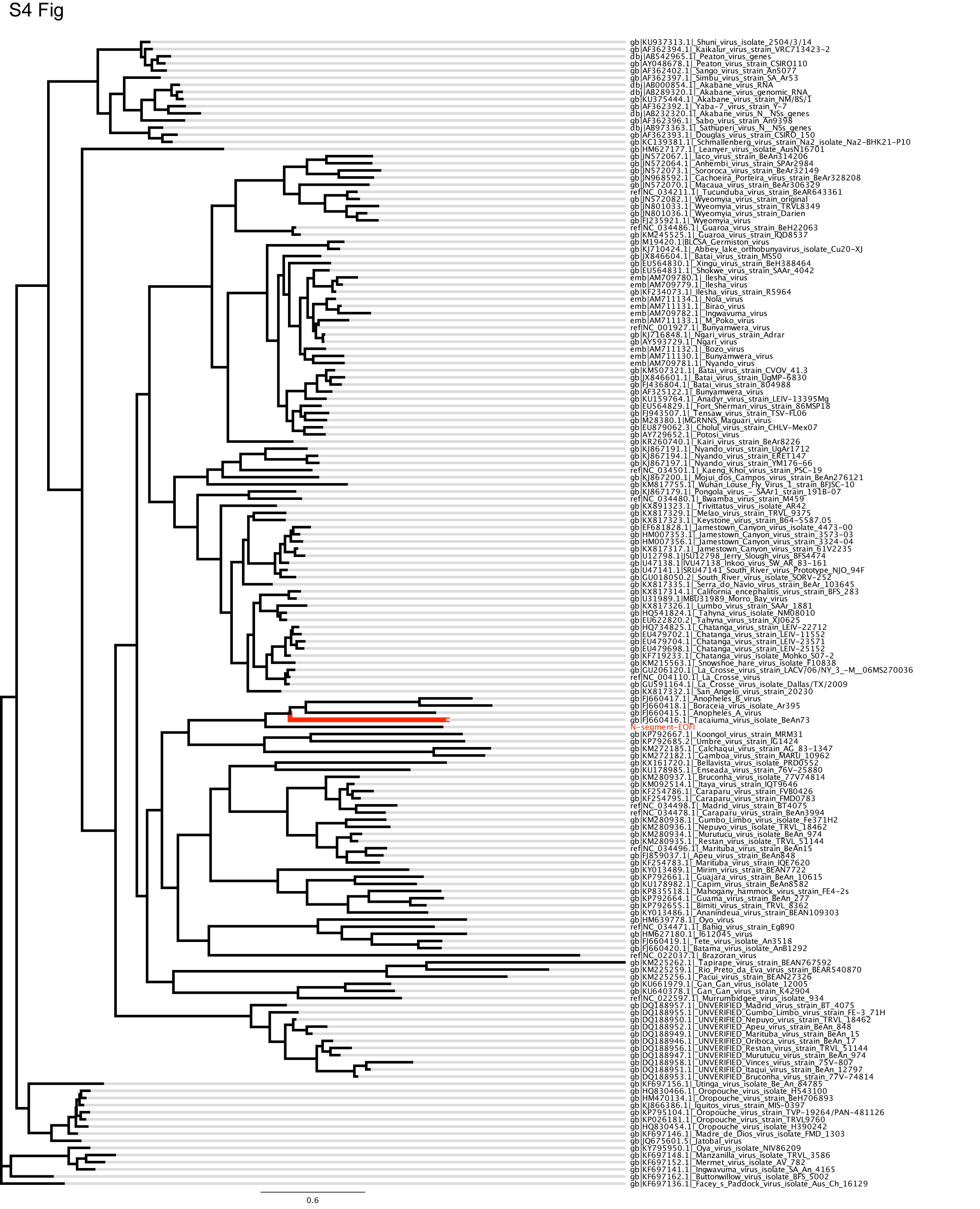

Supplement: S4 Fig — (TIF) [file pone.0218318.s004.tif]
