# Supplementary material for: Metagenomic next-generation sequencing of samples from pediatric febrile illness in Tororo, Uganda
Source: PLoS One. 2019 Jun 20;14(6):e0218318. doi: 10.1371/journal.pone.0218318 (PMC6586300; doi:10.1371/journal.pone.0218318)
Supplement: S1 Table — (A) Co-infection table for P. falciparum, (B) Co-infection table for HRV. (DOCX) [file pone.0218318.s005.docx]

**S1 Table:**

**(A)**

| **microbial co-infections with *P.falcipatum*** | **Number of cases** |
| --- | --- |
| Parvovirus B19 | 2 |
| Human herpesvirus 4 | 1 |
| Human immunodeficiency virus 1 | 1 |
| Norwalk virus | 1 |
| Orthobunyavirus | 1 |
| HRV-A | 1 |
| HRV-C | 1 |
| Rotavirus A | 1 |
| Parvovirus B19, HRV-C and human parechovirus 2 | 1 |

**(B)**

| **microbial co-infections with HRV** | **number of cases** |
| --- | --- |
| Human coronavirus OC43 | 2 |
| Human parainfluenza virus 1 | 2 |
| Rotavirus A | 2 |
| Hepatitis A | 1 |
| HHV type 5 | 1 |
| HHV type 6 | 1 |
| Human parainfluenza virus 4 | 1 |
| RSV | 1 |
| KI polyomavirus | 1 |
| RSV and Mamastrovirus 1 | 1 |
| RSV and human parainfluenza virus 1 | 1 |
| HHV type 5 HHV type 7, human coronavirus OC43 | 1 |
| Hepatitis B, human coronavirus NL63 and influenza A | 1 |
